# Streamlining sporozoite isolation from mosquitoes by leveraging the dynamics of migration to the salivary glands

**DOI:** 10.1186/s12936-022-04270-y

**Published:** 2022-09-13

**Authors:** Ashutosh K. Pathak, Justine C. Shiau, Blandine Franke-Fayard, Lisa M. Shollenberger, Donald A. Harn, Dennis E. Kyle, Courtney C. Murdock

**Affiliations:** 1 Department of Infectious Diseases, University of Georgia, Athens, GA 30602 USA; 2 Center for Tropical and Emerging Global Diseases, University of Georgia, Athens, GA 30602 USA; 3 Center for the Ecology of Infectious Diseases, University of Georgia, Athens, GA 30602 USA; 4 Department of Cellular Biology, University of Georgia, Athens, GA 30602 USA; 5 Odum School of Ecology, University of Georgia, Athens, GA 30602 USA; 6 Malaria Research Group, Department of Parasitology, Leiden University Medical Center, Leiden, The Netherlands; 7 Present Address: Department of Biological Sciences, Old Dominion University, Norfolk, VA 23529 USA; 8 Present Address: Department of Entomology, Comstock Hall, Cornell University, Ithaca, NY 14853 USA

**Keywords:** *Plasmodium berghei*, *Anopheles stephensi*, Oocysts, Sporozoites, Salivary glands, Density dependence, Extrinsic incubation period

## Abstract

**Background:**

Sporozoites isolated from the salivary glands of *Plasmodium*-infected mosquitoes are a prerequisite for several basic and pre-clinical applications. Although salivary glands are pooled to maximize sporozoite recovery, insufficient yields pose logistical and analytical hurdles; thus, predicting yields prior to isolation would be valuable. Preceding oocyst densities in the midgut is an obvious candidate. However, it is unclear whether current understanding of its relationship with sporozoite densities can be used to maximize yields, or whether it can capture the potential density-dependence in rates of sporozoite invasion of the salivary glands.

**Methods:**

This study presents a retrospective analysis of *Anopheles stephensi* mosquitoes infected with two strains of the rodent-specific *Plasmodium berghei.* Mean oocyst densities were estimated in the midguts earlier in the infection (11–15 days post-blood meal), with sporozoites pooled from the salivary glands later in the infection (17–29 days). Generalized linear mixed effects models were used to determine if (1) mean oocyst densities can predict sporozoite yields from pooled salivary glands, (2) whether these densities can capture differences in rates of sporozoite invasion of salivary glands, and (3), if the interaction between oocyst densities and time could be leveraged to boost overall yields.

**Results:**

The non-linear effect of mean oocyst densities confirmed the role of density-dependent constraints in limiting yields beyond certain oocyst densities. Irrespective of oocyst densities however, the continued invasion of salivary glands by the sporozoites boosted recoveries over time (17–29 days post-blood meal) for either parasite strain.

**Conclusions:**

Sporozoite invasion of the salivary glands over time can be leveraged to maximize yields for *P. berghei*. In general, however, invasion of the salivary glands over time is a critical fitness determinant for all *Plasmodium* species (extrinsic incubation period, EIP). Thus, delaying sporozoite collection could, in principle, substantially reduce dissection effort for any parasite within the genus, with the results also alluding to the potential for changes in sporozoites densities over time to modify infectivity for the next host.

**Supplementary Information:**

The online version contains supplementary material available at 10.1186/s12936-022-04270-y.

## Background

Preventing sporozoite establishment in the human liver has been a cornerstone of anti-malarial therapies for more than a century [1]. While the RTS/S vaccine underscored the potential for targeting the most devastating of human malaria, *Plasmodium falciparum,* its limited efficacy served as a reminder of the need for continual improvements to the vaccine design pipeline. Sporozoites isolated from the salivary glands of mosquitoes infected with human or rodent *Plasmodium* species offer several benefits that are either not possible, or difficult to assess with mosquito challenge models. Indeed, isolated sporozoites have served as starting points for identifying novel mechanisms of host-parasite interactions and developing pre-clinical assays for evaluating drugs and vaccines [2–12]. Developing approaches to maximize sporozoite yields from the salivary glands would help streamline existing workflows [13].

Despite the benefits of working with purified sporozoites, isolating sporozoites is a labour- and technique-intensive endeavour [2, 14]. The limited carrying capacity of salivary glands requires isolating sporozoites from large pools of salivary glands dissected from multiple mosquitoes [2–12]. However, low sporozoite yields present a hurdle to the design, execution, and interpretation of most assays. For instance, low yields may compel a researcher to reduce the number of study groups and/or replicates on an ad hoc basis at the cost of assay throughput and statistical power. Alternatively, yields could be fortified by dissecting more salivary glands, although low infection status and/or sporozoite densities in individuals may counter any net benefits gained from increasing dissection efforts. The ability to predict sporozoite yields a priori would be useful in overcoming these limitations. Following ingestion, parasites penetrate the mosquito midgut before establishing as oocysts, lining the epithelium under the basal lamina. Sporozoite replication is initiated in the oocysts from where they are released into the hemocoel before migrating to the salivary glands, ready for injection into another host. One commonly suggested predictor of sporozoite densities in the salivary glands is the density of oocysts in the midgut, where sporozoite replication occurs. Identifying a relationship between oocyst density and sporozoite yield would be beneficial as quantifying oocyst requires relatively little investment in labour and expertise [15, 16].

Previous studies investigating relationships between oocyst and sporozoite densities suggest the relationship is non-linear, with increasing oocyst densities generally associated with diminishing returns in sporozoite yields, likely due to density-dependent constraints on sporozoite production. These constraints could involve bottom-up and top-down processes limiting sporozoite production, such as increased competition for host resources, immune activation, or vector mortality [17–22]. Density-dependent effects on sporozoite replication could also have implications for the extrinsic incubation period (EIP) or the average time it takes for sporozoites to establish in the salivary glands. While differences in EIP are critical to *Plasmodium* fitness in general [23, 24], the potential for density-dependent asynchronicity in the dynamics of sporozoite migration is also supported by ecological theory, which documents similar density-dependent effects on the population growth rates of other organisms [25, 26]. In fact, changes in EIP with oocyst density might explain the lack of consensus in the literature regarding when (day post–blood meal) to recover *Plasmodium berghei* sporozoites, with times cited ranging from as early as 17 days to 29 days post–blood meal [2, 5, 17–19, 21, 27–38]. Thus, by accounting for shifts in the temporal dynamics of sporozoite invasion due to variation in oocyst burdens, the current study was undertaken to test the assumption that oocyst densities would predict optimal sporozoite yields.

Since its initial demonstration [39], mosquitoes infected with the rodent–specific *P. berghei* constitute an important component of the vaccine and drug discovery pipeline, with sporozoite yields from the salivary glands a critical bottleneck for several downstream applications [2]. Results described herein represent a retrospective, statistical analysis of a dataset consisting of 46 groups of mosquitoes infected with either one of the two strains of *P. berghei*, with the objective of asking the following questions: (1) how does oocyst density affect overall sporozoite yields, (2) can oocyst density predict rates of sporozoite migration, and (3) can this interaction be used to optimize sporozoite yields? Oocyst densities were estimated once between 11 and 15 days post-blood meal from a subgroup of individuals’ midguts, with sporozoites pooled from the salivary glands of another subgroup at one to four time points between 17 and 29 days post-blood meal (Additional file 3: Table S1). Sporozoite yields were then modelled with the (arithmetic) mean oocyst densities estimated earlier in the infection to determine whether yields were also dependent on time and/or parasite strain. The models were used to test the hypothesis that as oocyst densities increase, delayed replication and/or migration of sporozoites will enhance sporozoite yields over time, before eventually saturating and/or declining for both parasite strains. Analysis indicates that irrespective of parasite strain, sporozoite yields from pooled salivary glands were dependent on a non-linear relationship with the mean oocyst densities. In addition, sporozoite migration over the 17–29-day sampling period facilitated a linear increase in yields, albeit independent of the mean oocyst densities. Taken together, this study suggests sporozoite yields could be streamlined further by leveraging the dynamics of sporozoite migration. Finally, at the oocyst densities tested here, density-dependent migration may yet be the primary reason for increased sporozoite yields, although pooling salivary glands may only provide a qualitative overview of how sporozoite yields change over time; in other words, the rates of migration are likely to vary quantitatively between individuals, especially at oocyst densities where these rates could be used to maximize yields.

## Methods

### Chemical and consumables

Unless stated otherwise, all chemicals and consumables were purchased from Thermo Scientific Inc. (Hampton, NY).

### Parasite strains

Mice were infected with one of the two parasite strains, *P. berghei* WT (clone ANKA, referred to hereon as PbANKA, kind gift from Dr. Evlina Angov, Walter Reed Army Institute of Research, Silver Spring, MD), or a transgenic strain of *P. berghei*. Transgenic parasites were generated in female OF1 mice (6–7 weeks; Charles River, NL) in accordance with the European Guideline 86/609/EEC and follow the FELASA (Federation of European Laboratory Animal Science Associations) guidelines and recommendations concerning laboratory animal welfare. Animal experiments performed in Leiden University Medical Center (LUMC, Leiden, The Netherlands) were approved by the Animal Experiments Committee of the Leiden University Medical Centre (DEC 10099).

To generate the reporter line, GFP-Luc_ama1-eef1a_, the reference clone cl15cy1 of the *P. berghei* ANKA strain was used [40]. Two GFP-Luciferase expression cassettes were inserted into the neutral *p230p* gene locus (PBANKA_0306000) using standard transfection technologies (Additional file 1: Fig. S1a) [40]. To generate DNA construct pL1308, two standard DNA constructs were used, pL1063 [41, 42] and pL1156 [43]. The *eef1a-*GFP-Luc expression cassette of pL1063 (SacI fragment) was cloned into the SacI site of pL1165, generating plasmid pL1308 that contains the two *gfp-luc* fusion genes under the control of the *eef1a* (PBANKA_1133300) promoter or the *ama1* (PBANKA_0915000) promoter, respectively, and contains the two 5′ and 3′ *p230p* target regions (TR). The construct was linearized using SacII restriction sites outside of the 5′ and 3′ *p230p* TR before transfection. After transfection (exp. 1052), transfected parasites were obtained by flow-sorting based on GFP expression as described [41, 42], followed by cloning using the method of limiting dilution [40], resulting in line GFP-Luc_ama1-eef1a_ (line 1052cl1). Correct integration of DNA construct into the genome of GFP-Luc_ama1-eef1a_ was confirmed by Southern analyses of Pulsed Field Gel (PFG)-separated chromosomes (Additional file 1: Fig. S1b) [40]. PFG-separated chromosomes were hybridized with the 3’*utr Pbdhfr/ts* probe [40].

### Mice infections

All procedures described herein were approved by the University of Georgia’s Institutional Animal Care and Use Committee GA under Animal Use Protocol number A2016 06-010-Y1-A0 and A2020 01-013-Y2-A3. Three to 4 days before mosquito feeds, female C57BL/6 mice or Hsd:ICR(CD-1) mice (Envigo, Indianapolis, IN), aged 6–8 weeks, were injected intraperitoneally with 500 µL parasite suspended in sterile PBS with a density of 5 × 10^6^ or 10^7^ parasites per mouse with either *P. berghei* ANKA or the GFP-luciferase expressing strain of *P. berghei* (GFP-Luc_ama1-eef1a_, referred to herein as PbGFP-LUC_CON_). Total parasitaemia (trophozoites, schizonts, and gametocytes) was estimated starting 2 days post-infection with a Giemsa-stained smear of 1–2 µL of blood collected from the tail vein of each mouse [44]. Once total parasitaemia had reached 2–6% (usually 4 days), each mouse was anesthetized with ~ 0.5 ml of 1.25% 2,2,2-Tribromoethanol (v/v, Avertin, Sigma-Aldrich) and placed atop mosquito cages.

### Mosquito infections with *P. berghei* infected mice

*Anopheles stephensi* colonies were maintained as described previously, with adult mosquitoes maintained on a diet of 5% dextrose (w/v) and 0.05% para-aminobenzoic acid (w/v), a constant temperature of 27 °C and relative humidity of 75–85%, on a 12 h day/night cycle [44, 45]. Infections were performed with 3- to 7–day old, host-seeking female *An. stephensi* sorted into cages (17.5 cm L × 17.5 cm W × 17.5 cm H, BugDorm Inc., Taiwan). Approximately 24 h prior to infections, mosquitoes were transferred to a 20 °C chamber (Percival Scientific Inc., Dallas, IA) and starved before being fed on anesthetized mice for 15–20 min.

### Measuring oocyst densities in the midguts and sporozoite isolations

For quantifying oocyst densities in the midguts, mosquitoes from each group were vacuum aspirated directly into 70% ethanol, washed twice with ice-cold PBS, and oocysts enumerated as described previously [44, 45]. Salivary glands from mosquitoes in each group were used to isolate sporozoites, as described previously, with modifications [46]. Mosquitoes were first transferred to 16–ounce paper cups and cold anesthetized at – 20 °C for two to three minutes. Anesthetized mosquitoes were then transferred to a sterile petri dish (10 cm) maintained at − 2 to – 4 °C on a portable chill table (BioQuip Products, Rancho Dominguez, CA). Mosquitoes were surface sterilized with 70% ethanol before being dissected in chilled, sterile Schneider 2 (S2) insect cell culture media supplemented with 0.1% (v/v) fetal bovine serum. Using a tungsten micro–dissecting needle (0.25 mm diameter, 1 µm tip, Roboz Surgical Instrument Co., Gaithersburg, MD), salivary glands were pooled in a 1.5 ml microcentrifuge tube containing 100–500 µL of the dissection media; care was taken to ensure all six lobes were accounted for. Pooled glands were then disrupted with 5–10 passages through a sterile, 30G needle (Becton–Dickinson, Franklin Lakes, NJ) to release sporozoites [5]. Sporozoites were counted in a 10 μL aliquot with a haemocytometer and total yields were expressed as follows.

1 $$Y = (m \times 10) \times v$$

where *Y* denotes sporozoite yields, *m* indicates mean sporozoites counted from the four corner grids in a volume of 0.1 μL, with a constant of ‘10’ to further express ‘m’ per μL (i.e., 0.1 × 10 = 1 μL), and *v* indicates original volume of sporozoite suspension that the aliquot was drawn from (e.g., 500 μL). To express yields per mosquito, the following equation was used.

2 $$y = Y/n$$

where *y* denotes sporozoite yields per mosquito, *Y* indicates total sporozoite yields (from Eq. 1 above), with *n* representing the number of salivary glands pooled initially.

### Data analyses and statistical modelling

The dataset used in the current study represents 46 groups of *P. berghei*-infected mosquitoes, of which 36 were infected with *P. berghei* ANKA and the remaining 10 with PbGFP-LUC_CON_. Each group comprised 70–200 mosquitoes, distinguished based on being exposed to the same mouse (i.e., the same source of infection). Time of sampling was replicated over the 46 independent groups in a cross-sectional/partially nested sampling schedule where, for instance, sporozoite yields at 26 days post-blood meal were replicated across groups 4, 5, 17, 20, 27, 38, and 45 (Additional file 3: Table S1) [47, 48]. To accommodate the imbalanced nature of data collection, generalized linear mixed-effects models (GLMMs) were used to test hypotheses [47–50], with slopes of sporozoite yields dependent on the fixed effects/predictors (averaged oocyst densities, time, and/or parasite strain), but with intercepts allowed to vary randomly among the 46 groups (“fixed slopes, random intercepts”). Specifying groups as a random effect allows for the possibility that the relationship between oocyst and sporozoite densities measured in different individuals (since a mosquito can either be sampled for oocysts or sporozoites [2]) may show a greater correlation between mosquitoes that were exposed to the same source of infection (i.e., the same mouse) [18, 19, 21].

All data analyses and modelling were performed in RStudio [51], an integrated development environment for the open-source R package (version 4.1.0) [52], and associated packages. Graphical analyses were performed with the “ggplot2” package (version 3.3.5) [53] with the number of sporozoites recovered displayed as yields per mosquito (i.e., *y* from Eq. 2 above, rounded to the nearest integer). Because pooling prevents the estimation of each individual’s contribution of sporozoites, this method of expression does not represent a “true” average [54]. GLMMs were specified as suggested by the “glmmTMB” package [50]. Dispersion characteristics of residuals were tested in the “DHARMa” package (version 0.4.3). Tables were prepared for presentation with the “sjPlot” package (version 2.8.9) [55].

To maintain consistency with studies that rely on pooled sporozoites [2, 5, 17–19, 21, 27–38], statistical modelling was performed with sporozoite yields per mosquito (i.e., *y* from Eq. 2 above) specified as the dependent variable (model referred to herein as the ‘default’ model). However, to determine if differences in the number of salivary glands can alter model fit, a second model was built with total sporozoite yields expressed as the dependent variable (i.e., *Y* from Eq. 1 above), but with the number of salivary glands pooled added as an offset (*n* from Eq. 2 above) (model referred to herein as the ‘offset’ model). For instance, if 243,000 sporozoites were recovered from 31 mosquitoes, the dependent variable (*y*) in the ‘default model would be 7839 (or the quotient of 243,000/31, rounded to the nearest integer); the dependent variable in the ‘offset’ model would be 243,000, with the number of salivary glands dissected (i.e., 31) included as an ‘offset’ to account for differences in the number of salivary glands pooled between groups and/or time points [50]. Note that neither method of expression is based on true averages and thus, the ‘mean’ predicted by the statistical analysis should also not be considered a ‘true’ mean [55]. In addition, because of the distinct dependent variables, it was not possible to compare their fits with likelihood-based criteria [50].

Of the three fixed effects, parasite strain was coded as a categorical variable, while averaged oocyst densities and days post-blood meal were specified as continuous variables and centred and scaled with the mean and standard deviations, respectively [45]. Averaged oocyst densities were estimated by deriving the arithmetic mean of oocyst densities in all midguts, irrespective of infection status (i.e., midguts with oocysts ≥ 0). Expressing oocyst densities as the arithmetic mean was in keeping with how sporozoite yields were expressed, which, despite not being a true average, is more similar to how the arithmetic means (*y* in Eq. 2 above) are expressed, compared to other representations of central tendency [54]. Although only oocyst-positive mosquitoes will contribute to the sporozoite pool, our rationale for including all midguts irrespective of infection status is because salivary glands are generally dissected from all available mosquitoes irrespective of infection status (e.g., [2]).

In general, both ‘default’ and ‘offset’ models included sporozoite yields as a linear function of averaged oocyst densities, days post-blood meal (i.e., time), and parasite strain as main effects, and up to a three-way interaction. While a clear interaction between the three predictors would indicate differences between strains, a clear two-way interaction between oocyst densities and time on sporozoite density in the salivary glands would be indicative of density-dependence in sporozoite migration. To test the potential for saturation and decline in sporozoite yields due to mean oocyst densities and/or time (i.e., to model the hump-shaped effect on yields [45]), quadratic terms (or second-order polynomial) of the two predictors were also considered as additive (main) effects. However, in keeping with the objective of describing a simple predictive framework, automated stepwise elimination (based on likelihood-ratio tests and Akaike’s information criteria, corrected for small sample sizes (ΔAICc)) was used to identify the most parsimonious combination of predictors (predictors with p < 0.05) from either the ‘default’ or ‘offset’ models described above (referred to herein as ‘minimal’ models) [50]; note that distinct dependent variables meant direct statistical comparisons (using likelihood-based criteria) could not be performed ‘default’ and ‘offset’ models (either full or minimal) [50]. However, to enable indirect comparison, all visual displays of model fits were based on the minimal versions of ‘default’ and ‘offset’ models, with predicted means and 95% confidence intervals (95%CI) expressed per mosquito; predictions were derived via post-hoc simulations, as specified in the “ggeffects” package (version 1.1.1) [55].

## Results

### Summary of data

**Fig. 1:**
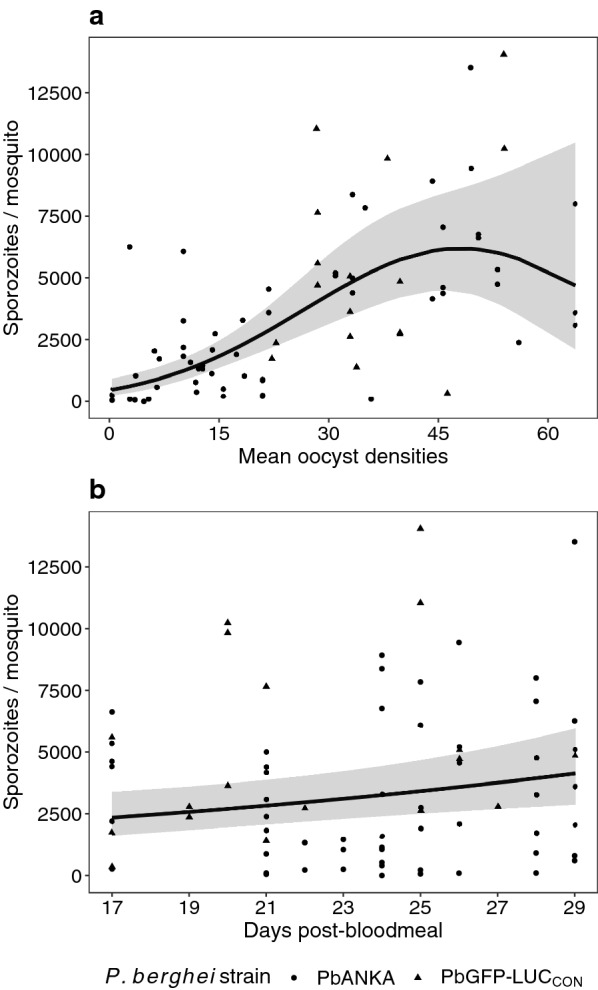
Sporozoite yields increase (**a**) non-linearly with (arithmetic) mean oocyst densities earlier in the infection (11–15 days post-blood meal), and (**b**) linearly over time (17–29 days post-blood meal) for mosquitoes infected with *P. berghei* strains PbANKA (circles) or PbGFP-LUC_CON_ (triangles). Lines and shaded areas indicate the mean and uncertainty (95% CI), respectively, predicted by the minimal model (Table 1). Mean oocyst densities were derived from counts of individual midguts, irrespective of infection status (oocysts ≥ 0). Note that although sporozoite yields are expressed per mosquito, values do not represent a true mean as counts are from pooled salivary glands, with no information of densities contributed by individuals

**Fig. 2:**
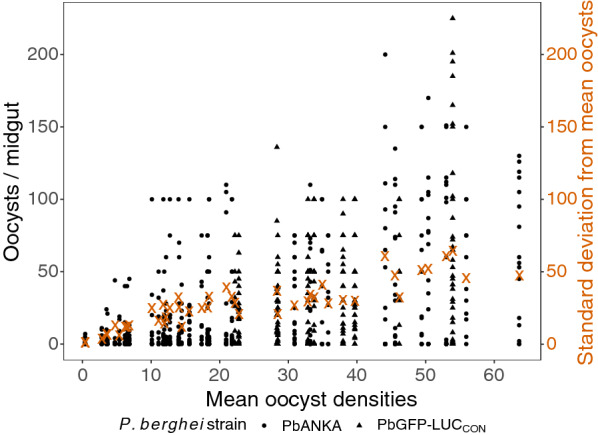
Mean oocyst densities for the 46 groups of mosquitoes increase linearly with the respective standard deviations (orange crosses), indicating the potential for collecting sporozoites from individuals with increasingly heterogeneous oocyst densities. Mosquitoes were infected with *P. berghei* strains PbANKA (circles) or PbGFP-LUC_CON_ (triangles)

The results are based on data from 2557 mosquitoes. Between 11 and 15 days post–blood meal, oocyst densities were quantified once from mosquitoes infected with *P. berghei* ANKA or PbGFP-LUC_CON_ (Additional file 4: Table S2). Starting 17 days post-blood meal, salivary glands from either a subsample or all remaining individuals were pooled to isolate sporozoites at one to four time points until 29 days post-blood meal (Fig. 1 and Additional file 3: Table S1). In general, heterogeneity (or differences) in oocyst densities between individuals, represented by the standard deviations, increased linearly with the corresponding mean for the 46 groups of mosquitoes (Pearson’s r (95%CI) = 0.88 (0.79–0.93), p < 0.001) (Fig. 2).

### Sporozoite yields increase non-linearly with mean oocyst densities, and linearly over time

The full model (Table 1) suggested sporozoite yields (*y* in Eq. 2) were driven by a non-linear relationship with the (arithmetic) mean of oocyst densities estimated earlier in the infection: the initial increase in yields (‘linear’, Z-value = 5.52, p < 0.001, Table 1) was eventually followed by a decline (‘quadratic’, Z-value = − 2.22, p = 0.027, Table 1) (Fig. 1a). Although the model was unable to identify a clear interaction between mean oocyst densities and time (Z-value = − 1.01, p = 0.312, Table 1), it suggested a relatively weaker (vs. oocyst densities), yet positive, linear effect of time on yields (Z-value = 2.62, p = 0.009, Table 1); in other words, at all mean oocyst densities tested here, the sporozoite migration dynamic resulted in higher sporozoite yields over time (Fig. 1b). Unlike the non-linear effect of mean oocyst densities (‘quadratic’), the model suggested yields did not decline with time post-infection (‘quadratic’, Z-value = 0.49, p = 0.623, Table 1). Additionally, the influence of mean oocyst densities and time on sporozoite yields did not differ between parasite strains (PbANKA or PbGFP-LUC_CON_) (rows 6, 8, 9, and 10, Table 1).

**Table 1:** The full and minimal model of sporozoite yields after stepwise elimination

Dependent variable (*y*) →		Sporozoites / salivary gland (‘mean sporozoite yields’)					
		Full model			Minimal model		
Row	Predictors	Z-value	*p*	Log-mean (se)	Z-value	*p*	Log-mean (se)
1	(Intercept)	54.17	**< 0.001**	7.85 (0.14)	66.63	**< 0.001**	7.91 (0.12)
2	Mean oocyst densities [linear]	5.52	**< 0.001**	6.15 (1.11)	6.17	**< 0.001**	5.97 (0.97)
3	Mean oocyst densities [quadratic]	− 2.22	**0.027**	− 2.57 (1.16)	− 2.83	**0.005**	− 2.81 (0.99)
4	Days post-bloodmeal [linear]	2.62	**0.009**	2.14 (0.82)	2.73	**0.006**	1.5 (0.55)
5	Days post-bloodmeal [quadratic]	0.49	0.623	0.28 (0.56)			
6	PbGFP-LUC_CON_ [vs PbANKA]	0.38	0.707	0.14 (0.37)			
7	Mean oocyst densities * Days post-bloodmeal [linear]	− 1.01	0.312	− 5.79 (5.73)			
8	PbGFP-LUC_CON_ * Mean oocyst densities [vs. PbANKA * Mean oocyst densities]	− 0.08	0.938	− 0.31 (4.00)			
9	PbGFP-LUC_CON_ * days post-bloodmeal [vs. PbANKA * days post-bloodmeal]	− 1.10	0.270	− 1.81 (1.64)			
10	PbGFP-LUC_CON_ * Mean oocyst densities * days post-bloodmeal [vs. PbANKA * Mean oocyst densities * days post-bloodmeal]	1.24	0.215	21.01 (16.95)			
Random effects							
11	*σ*_*ϵ*_^*2*^	0.27			0.28		
12	τ_00_ (groups)	0.37			0.34		
13	ICC (%)	0.57			0.55		
14	N (groups)	46			46		
15	Observations	75			75		
16	Marginal R^2^/Conditional R^2^ (%)	0.476/0.777			0.475/0.763		

Sporozoite yields are dependent on (arithmetic) mean oocyst densities and time. Yields do not represent a true average as sporozoites were recovered by pooling salivary glands without any information of densities in individuals, or infection status (sporozoites ≥ 0). For maintaining consistency with the latter, mean oocyst densities were estimated from midguts irrespective of infection status (i.e., oocysts ≥ 0)

While mean oocyst densities and time were able to explain 47.6% of the variation in the dataset (Marginal R^2^, Table 1), allowing random variation among groups in the contribution of these predictors improved the model’s account of the overall variation in the dataset (Conditional R^2^ = 77.7%, Table 1). Excluding group as a random effect significantly worsened model fit (ΔAICc = + 13.2). Finally, stepwise elimination of the least significant predictors from the full model (‘Minimal model’, Table 1) confirmed the non-linear effects of mean oocyst densities (‘linear’, Z-value = 6.17, p < 0.001 and ‘quadratic’, Z-value = − 2.83, p = 0.005) and time of sporozoite collection (i.e., days post-blood meal) were sufficient to model the overall trends (‘linear’, Z-value = 2.73, p = 0.006).

### Sporozoite yields increase with time, irrespective of how many salivary glands were pooled

**Fig. 3:**
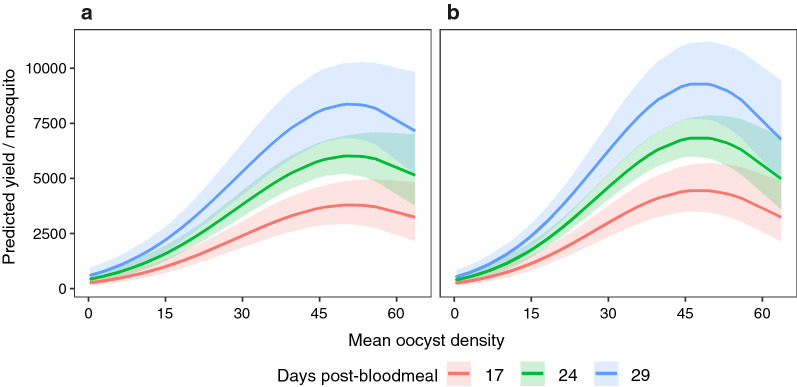
Predicted sporozoite yields over time (17, 24, and 29 days post-blood meal) at the respective mean oocyst densities (x-axis). Predictions after modelling sporozoite yields as (**a**) the ‘mean’ (minimal model of ‘default model’, Table 1) or (**b**) after considering differences in the number of salivary glands (minimal model of ‘offset model’, Additional file 5: Table S3). Lines and shaded areas indicate the mean and uncertainty (95%CI), respectively

In general, both the ‘default’ (Table 1) and ‘offset’ models (Additional file 5: Table S3) were consistent in highlighting the contribution of mean oocyst densities and time (rows 2, 3, and 4, Table 1 and Additional file 5: Table S3, differences in model structure are explained in ‘Data analyses and statistical modelling’ section). Sporozoite yields simulated over the same parameters for both models were also similar (Fig. 3); specifically, sporozoite yields increased with mean oocyst densities and time (colored lines), albeit with a corresponding increase in uncertainty (shaded areas, Fig. 3).

## Discussion

While mean oocyst densities estimated earlier in the infection were able to capture the non-linear trends in sporozoite recovery, the continued migration of sporozoites to the salivary glands also resulted in higher sporozoite yields over 17–29 days post-blood meal. The effect of mean oocyst densities and time were independent of parasite strain, and the number of salivary glands pooled, further suggesting that the effects described here should be broadly applicable. Taking the results together, this study describes a framework where mean oocyst densities estimated earlier in the infection could be used to approximate yields a priori, with the potential for yields to be maximized by leveraging the relationship between sporozoite yields and time. For example, at all mean oocyst densities, the ‘default’ model suggested that compared to 17 days post-blood meal, yields may be 1.3-, 1.7-, and 2.2-fold higher at 21, 25, and 29 days post-blood meal, respectively (Fig. 3). While yields will be dependent on mean oocyst densities, in general, accounting for the effect of time offer several benefits such as dissecting fewer mosquitoes, increasing the efficiency of otherwise cumbersome technical procedures, and potentially providing more reliability to downstream assays.

The range of sporozoites reported here generally corroborates previous findings from individual or pooled mosquitoes infected with *P. berghei* [17–19, 21, 33–38, 56–60]. The quadratic effect of mean oocyst densities on sporozoite yields increased with mean oocyst densities in a non-linear manner, wherein the initial increase in yields until ~ 50 oocysts were followed by a decline. In general, this non-linearity has been noted for sporozoites produced by both rodent and human *Plasmodium* species; current evidence suggests sporozoite densities may be dependent on complex interactions between nutritional availability, vector mortality, and immune responses to the oocysts and/or sporozoites [22, 61–63]. For *P. berghei*, the decline in sporozoite densities was noted at mean oocyst densities ≥ 50 per midgut [19, 21], with at least one study suggesting vector mortality during sporozoite egress as the most likely explanation for the reduced recovery [21]. A major benefit of this analysis is that the relationship between sporozoite yields and time post-infection can now be leveraged to reduce the risk of losing sporozoites at high mean oocyst densities (e.g., > 50) [19, 21], while also reducing dissection effort by boosting yields at lower oocyst densities. Although the model was unable to detect any evidence of a density-dependent delay (lack of clear association between mean oocyst densities and time), the clear effect of time suggested that the continued invasion of salivary glands should still be beneficial to sporozoite yields. Taken together, the effects of oocyst densities and time carry two implications for maximizing sporozoite yields. First, the non-linear effect of mean oocyst densities suggests that as oocyst densities increase (> 50), more mosquitoes would need to be dissected to obtain yields comparable to groups with lower densities. Second, although yields may increase linearly over time for all oocyst densities, groups with mean oocyst densities over 50 may still yield more sporozoites than groups with oocyst densities under 50, even if, for instance, sporozoite collection was delayed to 29 days post-blood meal (Fig. 3).

In addition to testing the potential for yields to decline with increasing mean oocyst densities (≥ 50), both models also tested whether yields would decline over time, by including a quadratic (“humped”) effect of days post-blood meal on sporozoite yields (Additional file 4: Table S2 and Additional file 5: Table S3). While the model was unable to identify a time point when yields started declining, simulations based on the model fit suggested that irrespective of mean oocyst density, yields became increasingly uncertain starting ~ 25 days post-blood meal (Figs. 1b and 2). Whether this was due to a lack of data, nutritional resources, heightened immune responses, a sign of deteriorating host health and increased mortality, or sporozoite senescence [19–21], is not possible to ascertain here. As such, until more data is available, results from the current study suggests sampling mosquitoes at ~ 25 days post-blood meal, but no later than 29 days when sporozoite infectivity has been suggested to decline [33]. Based on the available data, the model predicts 1.7-fold higher yields at 25 days (vs 17 days), irrespective of mean oocyst densities.

Together, mean oocyst densities and time were able to account for just under 50% of the overall variation in sporozoite yields from the dataset (e.g., Table 1). Allowing the contributions of the two predictors to vary randomly among groups significantly improved the model’s account of the total variation (R^2^ > 70%, Table 1), indicating the presence of other confounders specific to each group contributed significantly to the variation around the predicted yields (Figs. 1 and 3). For instance, of the groups where more information was available, one potential source of variation was a group of PbANKA-infected mosquitoes, which, despite only carrying a mean of 10.1 oocysts/midgut provided unexpectedly high sporozoite yields over time. However, only seven of the 17 individuals dissected (41%) showed evidence of having fed on the mouse (presence of eggs in ovaries during oocyst quantifications, mean feeding rates for all groups ± se = 72.4% ± 2.7), suggesting that < 50% of the group could contribute to the sporozoite pool (albeit with the caveat that estimation of feeding rates may be masked by eggs being reabsorbed by the mother [64–66]). These seven blood-fed individuals were carrying a mean of 25 oocysts per midgut, which was higher than the 10.1 calculated earlier, but more likely to explain the higher sporozoite yields observed for the group. In contrast, a group of PbGFP-LUC_CON_-infected mosquitoes with a mean oocyst density of 46.2 should have yielded, according to the model, significantly more sporozoites than the 5250 recovered from 15 salivary glands (or 350 per mosquito) at 17 days post-blood meal. To determine why the yields were low, oocysts were quantified in the midguts of these 15 individuals at the time of sporozoite collections (i.e., 17 days) and found to be carrying 17 mean oocysts per midgut, which was lower than the 46.2 mean oocysts measured earlier from the same group. Whether the reduction in oocyst densities were due to excess mortality in the cage or overestimation of mean oocyst densities earlier, the low oocyst densities were the likely reason for the low yields observed. While the models identified these groups as sources of variation, in general, they were consistent with the conclusion that yields were driven by mean oocyst densities and time.

While the instances above highlight how ‘accurate’ estimation of mean oocyst densities is also critical to predicting sporozoite yields over time, despite the consistency, the effect of time on sporozoite yields was less clear (Table 1). Future experiments with sporozoites collected over a more balanced sampling schedule, and a fixed number of individuals could address some of the study limitations and offer clearer insight into whether rates of sporozoite migration do indeed vary with oocyst density. As oocyst densities increase, sporozoite migration over time may be the primary reason for increased yields (Fig. 1a), however, a clear estimation of its contribution may be confounded by pooling sporozoites from individuals with increasingly heterogenous oocyst densities (shaded areas, Additional file 2: Fig. S2).

Further, this heterogeneity between individuals in their contribution to the sporozoite pool could explain why adjusting for differences in the number of salivary glands did not identify a clearer effect of time (Additional file 5: Table S3). Thus, estimating the relationship between oocyst density and sporozoite yields from pooled salivary glands likely only provides qualitative insight into a process that may vary quantitatively among individuals. Considering this, isolating sporozoites from all mosquitoes at a single day post-blood meal (e.g., 25–26 days), instead of at fixed intervals, may be a more efficient use of mosquitoes and time, while still achieving maximal sporozoite yields.

There are two additional caveats associated with the study and analysis. First, although the main effects of mean oocyst densities and time were common to both parasite strains, the objective of this study was not to compare strains and as such, the potential for strains to differ in overall sporozoite output should not be disregarded [21]. Second, differences between mosquito colonies due to genetic (e.g., *An. stephensi* “Indian” vs “SDA–500”) [67, 68] and/or epigenetic effects (for instance, larval culture conditions and its carry-over effect on adults) [69], may manifest as differences in parasite infection, replication, and sporozoite yields. While these differences suggest that more careful consideration may be necessary before settling on specific (or range of) parameters, the fundamental nature of the relationship between oocyst densities, time, and sporozoite yields should be consistent.

## Conclusions

As with other organismal systems, density-dependent effects in *Plasmodium* parasites resulted in a non-linear relationship between the density of parasites successfully establishing (oocysts) and the transmission stages (sporozoites) [23, 70]. In general, the temporal dynamics of sporozoite migration are critical to *Plasmodium* fitness. Thus, in addition to indicating how prior oocyst densities could be leveraged to streamline sporozoite isolations for other *Plasmodium* species [2], the current study highlights the potential for oocyst densities to predict when a mosquito becomes infectious (EIP), while also suggesting how changes in sporozoite densities over time can alter parasite infectivity to the next host [33, 71]. Irrespective of the downstream application, the versatility offered by rodent malaria models underscores their continued utility in evaluating strategies to manage parasite transmission, but also how these efforts can be aided by an increased understanding of fundamental aspects of vector-parasite interactions.

## Supplementary Information

**Additional file 1: Figure S1.** Generation and genotyping of the transgenic *P. berghei* ANKA reporter line GFP-Luc_ama1-eef1a_ (1052cl1). (a) Schematic representation of the generation of GFP-Luc_ama1-eef1a_, (line 1052cl1) obtained after transfection by flow- sorting. DNA construct pL1308 is linearized at the SacII sites and integration occurs by double cross-over into the neutral *p230p* locus on chromosome (chr) 3. The construct contains two reporter expression cassettes, both containing the *gfp-luciferase* fusion gene under the control of either the schizont-specific *ama1* promoter or the constitutive *eef1a* promoter. (b) Southern analysis of PFG-separated chromosomes confirms integration of the DNA construct pL1308 into the *p230p* locus on chr 3 of GFP-Luc_ama1-eef1a_ (1052cl1). The chromosomes are hybridized using a probe recognizing the 3′*utr Pbdhfr/ts* of the SM of the integrated construct which also hybridizes to the endogenous Pbdhfr/ts (PBANKA_0719300) on chr 7; control: the reference clone cl15cy1 of the *P. berghei* ANKA strain.

**Additional file 2: Figure S2.** Increasing yields predicted by (a) mean oocyst densities (yellow line) correspond with increasing uncertainty (yellow shaded area). (b) In groups with higher mean oocyst densities (e.g., 45), estimating the contribution of time may be difficult because of the possibility of pooling individuals with heavily infected midgut (~ 50 oocysts, left pane) consisting of some oocysts that have contributed sporozoites already (arrows) and some still in the process of doing so (arrowheads), with another individual with low infected midgut (4 oocysts, right pane) where the entire contingent of sporozoites have been released (arrowheads). Images were taken at 400 × magnification at 26 days post-blood meal, from individuals whose salivary glands were combined into the same pool.

**Additional file 3: Table S1**. Sampling regime for collecting sporozoites from 46 groups of mosquitoes, infected with two strains of *P. berghei*, over time (17–29 days post-blood meal); note salivary glands were not collected from any group at 18 days post-blood meal. Checkmarks indicate when sample collections were performed between 17 and 29 days post-blood meal from the respective groups; multiple check marks for the same group depict when sporozoites were collected for that group.

**Additional file 4: Table S2.** Sample sizes and measures of parasite infection in the midguts.

**Additional file 5: Table S3**. The full and minimal versions of the ‘offset’ model of sporozoite yields to adjust for differences in the number of salivary glands sampled (also see Table 1 for the ‘default’ model, and ‘Data analyses and statistical modelling’ section for rationale).

## Data Availability

Upon acceptance, all data and statistical code from the study will be posted to a public repository.
