# Supplementary figures and images for: Streamlining sporozoite isolation from mosquitoes by leveraging the dynamics of migration to the salivary glands
Source: Malar J. 2022 Sep 13;21:264. doi: 10.1186/s12936-022-04270-y (PMC9472382; doi:10.1186/s12936-022-04270-y)

**a**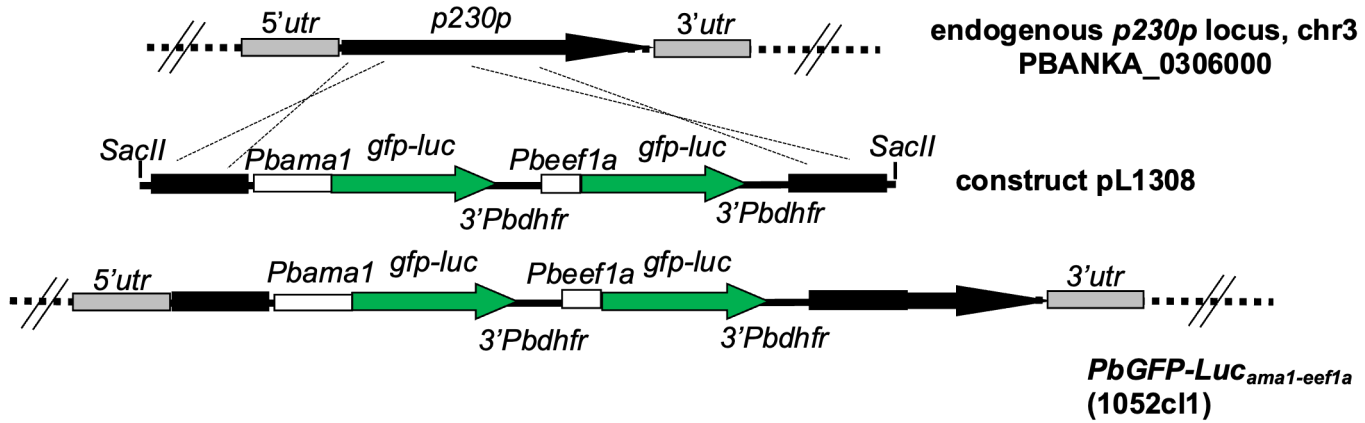**b**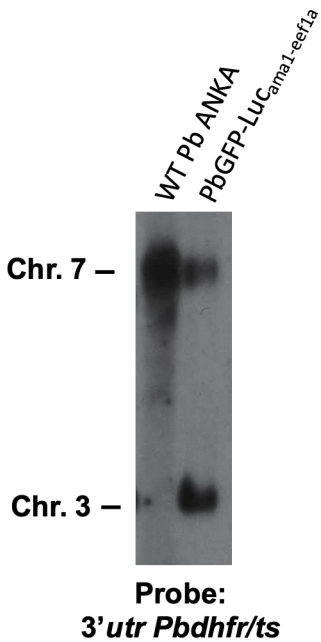

Supplement: Supplementary file 1 — Additional file 1: Figure S1. Generation and genotyping of the transgenic P. berghei ANKA reporter line GFP-Lucama1-eef1a (1052cl1). (a) Schematic representation of the generation of GFP-Lucama1-eef1a, (line 1052cl1) obtained after transfection by flow- sorting. DNA construct pL1308 is linearized at the SacII sites and integration occurs by double cross-over into the neutral p230p locus on chromosome (chr) 3. The construct contains two reporter expression cassettes, both containing the gfp-luciferase fusion gene under the control of either the schizont-specific ama1 promoter or the constitutive eef1a promoter. (b) Southern analysis of PFG-separated chromosomes confirms integration of the DNA construct pL1308 into the p230p locus on chr 3 of GFP-Lucama1-eef1a (1052cl1). The chromosomes are hybridized using a probe recognizing the 3′utr Pbdhfr/ts of the SM of the integrated construct which also hybridizes to the endogenous Pbdhfr/ts (PBANKA_0719300) on chr 7; control: the reference clone cl15cy1 of the P. berghei ANKA strain. [file 12936_2022_4270_MOESM1_ESM.pdf]

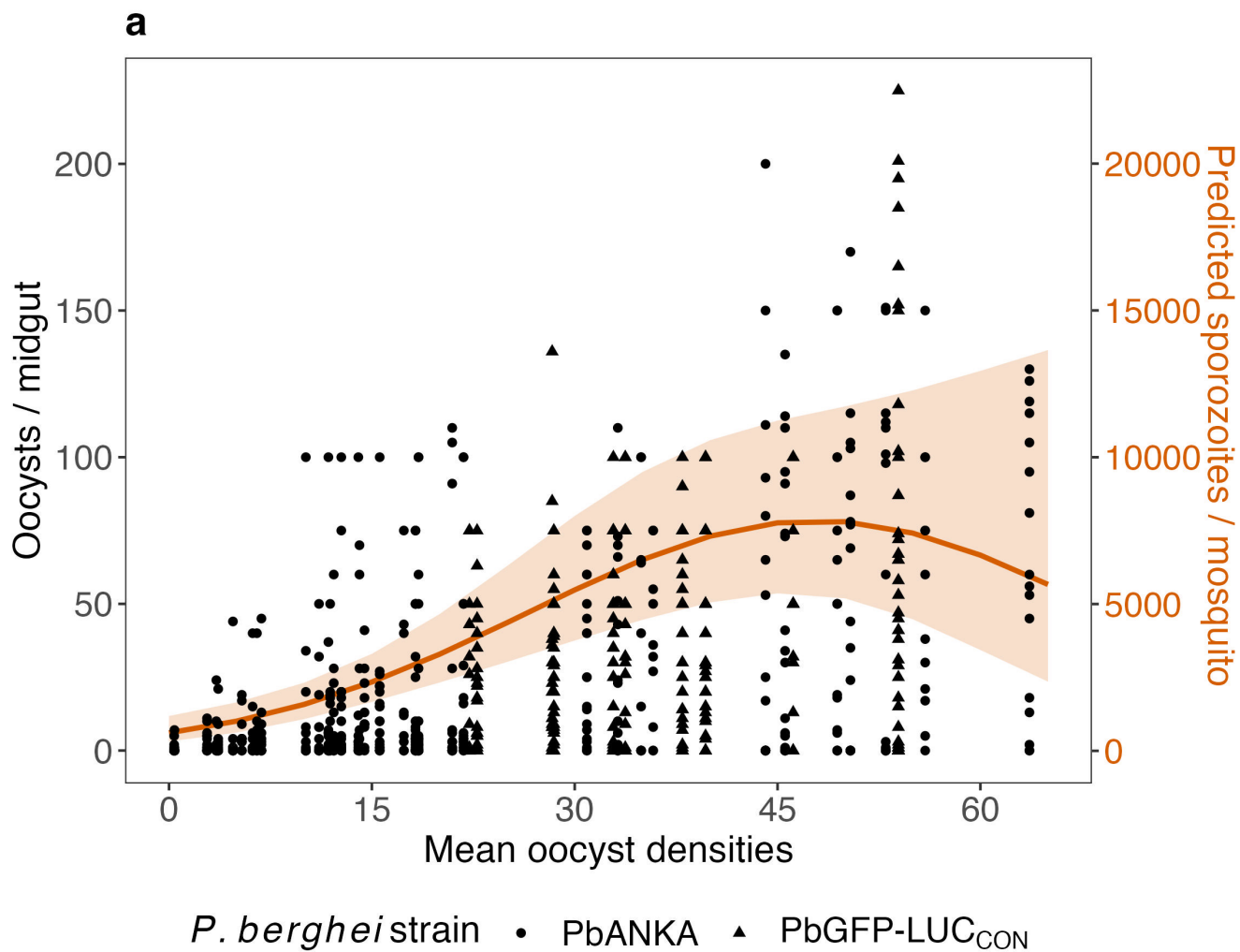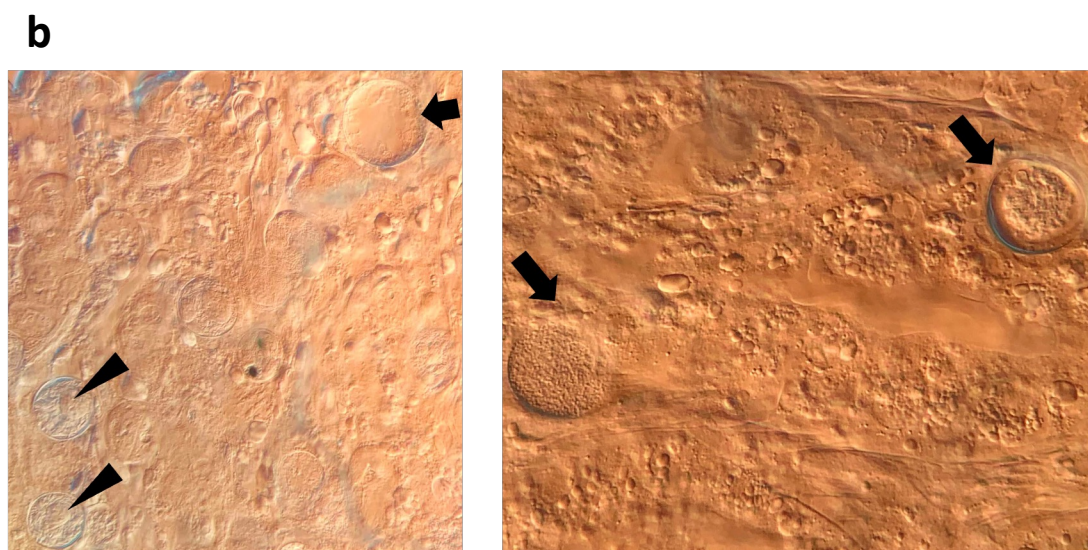

Supplement: Supplementary file 2 — Additional file 2: Figure S2. Increasing yields predicted by (a) mean oocyst densities (yellow line) correspond with increasing uncertainty (yellow shaded area). (b) In groups with higher mean oocyst densities (e.g., 45), estimating the contribution of time may be difficult because of the possibility of pooling individuals with heavily infected midgut (~ 50 oocysts, left pane) consisting of some oocysts that have contributed sporozoites already (arrows) and some still in the process of doing so (arrowheads), with another individual with low infected midgut (4 oocysts, right pane) where the entire contingent of sporozoites have been released (arrowheads). Images were taken at 400 × magnification at 26 days post-blood meal, from individuals whose salivary glands were combined into the same pool. [file 12936_2022_4270_MOESM2_ESM.pdf]
