# Supplementary material for: Streamlining sporozoite isolation from mosquitoes by leveraging the dynamics of migration to the salivary glands
Source: Malar J. 2022 Sep 13;21:264. doi: 10.1186/s12936-022-04270-y (PMC9472382; doi:10.1186/s12936-022-04270-y)
Supplement: Supplementary file 3 — Additional file 3: Table S1. Sampling regime for collecting sporozoites from 46 groups of mosquitoes, infected with two strains of P. berghei, over time (17–29 days post-blood meal); note salivary glands were not collected from any group at 18 days post-blood meal. Checkmarks indicate when sample collections were performed between 17 and 29 days post-blood meal from the respective groups; multiple check marks for the same group depict when sporozoites were collected for that group. [file 12936_2022_4270_MOESM3_ESM.docx]

| **Supplementary table 1** | | | | | | | | | | | | | |
| --- | --- | --- | --- | --- | --- | --- | --- | --- | --- | --- | --- | --- | --- |
| **Parasite strain** | **Group**  **number** | **Days post-bloodmeal** | | | | | | | | | | | |
|  |  | 17 | 19 | 20 | 21 | 22 | 23 | 24 | 25 | 26 | 27 | 28 | 29 |
| PbANKA | 1 |  |  |  |  |  |  |  | ✓ |  |  |  |  |
| PbANKA | 2 |  |  |  | ✓ |  |  |  |  |  |  |  |  |
| PbANKA | 3 |  |  |  |  |  |  |  |  |  |  |  | ✓ |
| PbANKA | 4 |  |  |  |  |  |  |  |  | ✓ |  |  | ✓ |
| PbANKA | 5 |  |  |  |  |  |  |  |  | ✓ |  |  |  |
| PbANKA | 6 | ✓ |  |  | ✓ |  |  |  | ✓ |  |  | ✓ |  |
| PbANKA | 7 |  |  |  |  |  | ✓ |  |  |  |  |  |  |
| PbGFP-LUC_CON_ | 8 | ✓ |  |  |  |  |  |  |  |  |  |  |  |
| PbGFP-LUC_CON_ | 9 |  |  | ✓ |  |  |  |  |  |  |  |  |  |
| PbANKA | 10 |  |  |  | ✓ |  |  |  |  |  |  |  |  |
| PbANKA | 11 |  |  |  |  | ✓ | ✓ |  |  |  |  |  |  |
| PbANKA | 12 |  |  |  | ✓ |  |  |  |  |  |  |  |  |
| PbANKA | 13 |  |  |  |  | ✓ |  |  |  |  |  |  |  |
| PbANKA | 14 |  |  |  |  |  |  |  | ✓ |  |  |  |  |
| PbANKA | 15 |  |  |  |  |  | ✓ |  |  |  |  |  |  |
| PbANKA | 16 |  |  |  |  |  |  | ✓ |  |  |  |  |  |
| PbGFP-LUC_CON_ | 17 |  |  | ✓ |  |  |  |  | ✓ | ✓ |  |  |  |
| PbANKA | 18 |  |  |  |  |  |  | ✓ |  |  |  |  |  |
| PbANKA | 19 |  |  |  |  |  |  | ✓ |  |  |  |  |  |
| PbANKA | 20 |  |  |  |  |  |  |  |  | ✓ |  |  | ✓ |
| PbANKA | 21 |  |  |  | ✓ |  |  |  |  |  |  | ✓ | ✓ |
| PbANKA | 22 |  |  |  |  |  |  |  | ✓ |  |  |  |  |
| PbANKA | 23 |  |  |  |  | ✓ |  | ✓ |  |  |  |  |  |
| PbANKA | 24 | ✓ |  |  |  |  |  |  |  |  |  | ✓ |  |
| PbGFP-LUC_CON_ | 25 |  |  |  |  |  |  |  | ✓ |  |  |  |  |
| PbANKA | 26 |  |  |  | ✓ |  |  | ✓ |  |  |  |  |  |
| PbGFP-LUC_CON_ | 27 | ✓ |  |  | ✓ |  |  |  |  | ✓ |  |  |  |
| PbANKA | 28 |  |  |  |  |  |  | ✓ |  |  |  |  |  |
| PbANKA | 29 |  |  |  |  |  |  |  |  |  |  | ✓ |  |
| PbANKA | 30 |  |  |  |  |  |  |  |  |  |  |  | ✓ |
| PbANKA | 31 | ✓ |  |  | ✓ |  |  | ✓ |  |  |  |  |  |
| PbANKA | 32 |  |  |  |  |  |  |  |  |  |  |  | ✓ |
| PbANKA | 33 | ✓ |  |  | ✓ |  |  |  |  |  |  | ✓ |  |
| PbGFP-LUC_CON_ | 34 |  |  |  | ✓ |  |  |  |  |  |  |  |  |
| PbGFP-LUC_CON_ | 35 |  | ✓ |  |  | ✓ |  |  |  |  | ✓ |  | ✓ |
| PbANKA | 36 | ✓ |  |  | ✓ |  |  |  | ✓ |  |  | ✓ |  |
| PbANKA | 37 |  |  |  |  |  |  | ✓ |  |  |  |  |  |
| PbANKA | 38 |  |  |  |  |  |  |  |  | ✓ |  |  | ✓ |
| PbANKA | 39 | ✓ |  |  |  |  |  | ✓ |  |  |  |  |  |
| PbANKA | 40 |  |  |  |  |  |  |  | ✓ |  |  |  |  |
| PbANKA | 41 |  |  |  |  |  |  |  |  |  |  | ✓ |  |
| PbGFP-LUC_CON_ | 42 | ✓ |  |  |  |  |  |  |  |  |  |  |  |
| PbANKA | 43 |  |  |  |  |  |  | ✓ |  |  |  |  |  |
| PbGFP-LUC_CON_ | 44 |  |  | ✓ |  |  |  |  | ✓ |  |  |  |  |
| PbANKA | 45 |  |  |  |  |  |  |  |  | ✓ |  |  | ✓ |
| PbGFP-LUC_CON_ | 46 |  | ✓ |  |  |  |  |  |  |  |  |  |  |
| Number of data points | | 9 | 2 | 3 | 11 | 4 | 3 | 10 | 8 | 7 | 1 | 7 | 9 |
