# Supplementary material for: Streamlining sporozoite isolation from mosquitoes by leveraging the dynamics of migration to the salivary glands
Source: Malar J. 2022 Sep 13;21:264. doi: 10.1186/s12936-022-04270-y (PMC9472382; doi:10.1186/s12936-022-04270-y)
Supplement: Supplementary file 4 — Additional file 4: Table S2. Sample sizes and measures of parasite infection in the midguts. [file 12936_2022_4270_MOESM4_ESM.docx]

| **Supplementary table 2** | | |
| --- | --- | --- |
| **Strain** | **PbANKA** | **PbGFP-LUC_CON_** |
| **Groups** | 36 | 10 |
| **Total midguts dissected (range)** | 537 (9-23) | 270 (13-46) |
| **Mean oocyst densities (± se, range)** | 21.2 (± 3, 0.38 - 63.6) | 34.6 (± 3.2, 22.2 - 53.9) |
| **Mean oocyst prevalence (± se, range)** | 65.8 (± 3.1, 10 - 91.7) | 81.2 (± 3.5, 57.1 - 91.3) |
| **Total salivary glands dissected (range)** | 1125 (10 - 43) | 625 (10 - 133) |
