# Supplementary material for: Streamlining sporozoite isolation from mosquitoes by leveraging the dynamics of migration to the salivary glands
Source: Malar J. 2022 Sep 13;21:264. doi: 10.1186/s12936-022-04270-y (PMC9472382; doi:10.1186/s12936-022-04270-y)
Supplement: Supplementary file 5 — Additional file 5: Table S3. The full and minimal versions of the ‘offset’ model of sporozoite yields to adjust for differences in the number of salivary glands sampled (also see Table 1 for the ‘default’ model, and ‘Data analyses and statistical modelling’ section for rationale). [file 12936_2022_4270_MOESM5_ESM.docx]

| Supplementary table 3 | | | | | | | |
| --- | --- | --- | --- | --- | --- | --- | --- |
| Dependent variable (*y*) → | | **Total sporozoites (offset = number of salivary glands)** | | | | | |
|  | | **Full model** | | | **Minimal model** | | |
| *Row* | ***Predictors*** | *Z-value* | *p* | *Log-mean (se)* | *Z-value* | *p* | *Log-mean (se)* |
| 1 | (Intercept) | 57.55 | **<0.001** | 7.88 (0.14) | 72.21 | **<0.001** | 7.95 (0.11) |
| 2 | Mean oocyst densities [linear] | 6.02 | **<0.001** | 6.37 (1.06) | 6.94 | **<0.001** | 6.19 (0.89) |
| 3 | Mean oocyst densities [quadratic] | -2.52 | **0.012** | -2.73 (1.08) | -3.16 | **0.002** | -2.88 (0.91) |
| 4 | Days post-bloodmeal [linear] | 2.37 | **0.018** | 2.05 (0.86) | 2.46 | **0.014** | 1.42 (0.58) |
| 5 | Days post-bloodmeal [quadratic] | 0.38 | 0.703 | 0.23 (0.60) |  |  |  |
| 6 | PbGFP-LUC_CON_ [vs PbANKA] | 0.27 | 0.790 | 0.09 (0.33) |  |  |  |
| 7 | Mean oocyst densities * Days post-bloodmeal [linear] | -0.93 | 0.351 | -5.64 (6.04) |  |  |  |
| 8 | PbGFP-LUC_CON_ * Mean oocyst densities [vs. PbANKA * Mean oocyst densities] | 0.07 | 0.943 | 0.25 (3.54) |  |  |  |
| 9 | PbGFP-LUC_CON_ * days post-bloodmeal [vs. PbANKA * days post-bloodmeal] | -1.38 | 0.168 | -2.19 (1.59) |  |  |  |
| 10 | PbGFP-LUC_CON_ * Mean oocyst densities * days post-bloodmeal [vs. PbANKA * Mean oocyst densities * days post-bloodmeal] | 1.56 | 0.118 | 24.17 (15.47) |  |  |  |
|  | **Random Effects** | | | | | | |
| 11 | σ^2^ | 0.21 | | | 0.22 | | |
| 12 | τ_00_ (group) | 0.30 | | | 0.26 | | |
| 13 | ICC | 0.59 | | | 0.54 | | |
| 14 | N (groups) | 46 | | | 46 | | |
| 15 | Observations | 75 | | | 75 | | |
| 16 | Marginal R^2^ / Conditional R^2^ | 0.553 / 0.817 | | | 0.558 / 0.797 | | |
